# Improving Electrochemical Performance in Planar On‐Chip Zn‐ion Micro‐Batteries via Interlayer Strategies

**DOI:** 10.1002/smll.202405733

**Published:** 2024-10-14

**Authors:** Yijia Zhu, Nibagani Naresh, Xiaopeng Liu, Jingli Luo, Yujia Fan, Mengjue Cao, Bing Li, Mingqing Wang, Buddha Deka Boruah

**Affiliations:** ^1^ Institute for Materials Discovery University College London London WC1E 7JE UK

**Keywords:** high‐performance, interlayer strategy, micro‐electrodes, planar Zn‐ion micro‐batteries

## Abstract

The imperative development of planar on‐chip micro‐batteries featuring high‐capacity electrodes and environmentally safer, cost‐effective, and stable systems is crucial for powering forthcoming miniaturized systems‐on‐chip smart devices. However, research in the area of high‐stability micro‐batteries is limited due to the complex fabrication process, the stability of micro‐electrodes during cycling, and the challenge of maintaining higher capacity within a limited device footprint. In response to this need, this study focuses on providing highly stable and high‐capacity micro‐electrodes. This involves adding a PEDOT layer between the electrode material and the current collector, applied within a planar polyaniline cathode and zinc anode device structure to enhance charge storage performance. This straightforward strategy not only improves device stability over long‐term cycling and reduces charge transfer resistance but also increases charge storage capacities from 17.64 to 19.75 µAh cm^−^^2^ at 0.1 mA cm^−^^2^. Consequently, the Zn‐ion micro‐batteries achieve notable peak areal energy and power of 18.82 µWh cm^−^^2^ and 4.37 mW cm^−^^2^, respectively. This work proposes an effective strategy to enhance the electrochemical performance of planar micro‐batteries, a critical advancement for the development of advanced portable electronics.

## Graphical abstract

The advancement of planar on‐chip micro‐batteries with high‐capacity electrodes, as well as systems that are cost‐effective, environmentally friendly, and stable, is essential for the next generation of miniaturized systems‐on‐chip smart technologies. One promising approach to improving charge storage performance is incorporating an interfacial PEDOT layer between the electrode material and the current collector. This technique, used in a planar design with a polyaniline cathode and zinc anode, optimizes the device's overall efficiency.

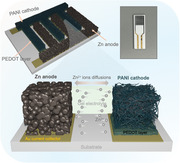

## 1 Introduction

The advent of the Internet of Things (IoT) has made miniaturization and integration key trends in the evolution of microelectronics. Significant strides have been made in developing microelectronic devices for wearables and implants, including micro‐robots and micro‐sensors, which are poised to become integral to our daily lives. These compact devices are highly effective in tasks such as data processing and wireless signal transmission within spaces smaller than a few cubic millimeters, showing great potential in health monitoring, medical diagnosis, and disease treatment.^[^ ^1^ ^]^ For these devices to operate efficiently, a reliable energy supply is essential. As the size of wearable and implantable microelectronics continues to decrease while their capabilities increase, there is a growing need for corresponding micro‐power sources, such as micro‐batteries, that can deliver substantial energy outputs.^[^ ^2^, ^3^ ^]^ Conventional micro‐battery structures, which resemble layered sandwiches with positive and negative electrodes separated by separators, present various challenges. Thin electrodes have limited energy density, while three (3D) thick electrodes suffer from slow ion diffusion. Moreover, the use of 3D thick electrodes with specific patterns requires precise alignment.^[^ ^4^ ^]^ A promising alternative is the planar‐type device configuration, where electrodes are arranged in a planar pattern on the same substrate, creating a flat device structure. This configuration offers several benefits, including improved control over crucial battery attributes such as internal resistance and ionic diffusion distance, all without the need for a separator. Most notably, it provides a practical solution for reducing battery size and allows for seamless integration with on‐chip microelectronic devices.

Among competitive energy storage systems, Li‐based micro‐batteries are prominent due to their high energy density, lightweight, and long‐term cycling stability. However, there are significant safety concerns with Li‐ion micro‐batteries, including the risk of thermal runaway.^[^ ^5^ ^]^ Additionally, their production requires sophisticated environments, which can increase costs when integrating these batteries with other microelectronics for system‐on‐chip smart devices. In contrast, Zn‐based micro‐batteries (ZIMBs) are promising candidates due to their cost‐efficiency, safety, and abundance of materials compared to lithium‐ion batteries.^[^ ^4^, ^6^, ^7^ ^]^ The zinc anode offers high capacity (820 mAh g^−1^, 5855 mAh cm^−^^3^) and a low redox potential (−0.76 V vs. SHE), making it possible to use aqueous electrolytes.^[^ ^8^ ^]^ This allows for high‐capacity cathode materials that are more stable in air, simplifying the assembly process and reducing manufacturing difficulties. Recent research has explored various cathode materials, such as manganese‐based, vanadium‐based, and Prussian blue analogs materials.^[^ ^8^ ^]^ However, many of these cathode materials degrade over time and have small interlayer distances, leading to limited long‐term durability, unstable efficiency, and low capacity.^[^ ^9^, ^10^, ^11^ ^]^ Polymer‐based cathode materials have emerged as significant candidates due to their stability and environmental friendliness. For example, Sun et al. reported a reversible battery by polymerizing 1, 2, 4, 5‐tetraaminobenzene and pyrene‐4, 5, 9, 10‐tetraone compounds together to reduce binding‐H_2_O ability and increase structural stability^[^ ^12^ ^]^. However, these strategies are typically applied in conventional Zn‐ion batteries by coating the synthesized material on current collectors, with limited attention to their application in planar ZIMBs. Additionally, the peeling off of active materials during cycling is a critical issue for micro‐electrodes that still needs to be addressed, along with the exploration of high‐capacity materials. Therefore, comprehensive strategies are essential to mitigate these issues and extend the operational lifespan of high‐performance, environmentally friendly planar ZIMBs.

In our study, we investigated approaches to enhance the capacity and stability of PANI‐based cathode materials loaded onto microelectrodes for testing against zinc micro‐anodes in ZIMBs. This involved adding a PEDOT layer between the current collector and the PANI cathode material, followed by stabilizing the system with a gel electrolyte. As anticipated, the ZIMBs exhibited a remarkable areal capacity of 20.5 µAh cm^−^^2^ at 0.05 mA cm^−^^2^, coupled with cycling stability and a capacity retention of 89% even after 2000 cycles. Additionally, the efficiency of these ZIMBs yielded a high areal energy of 18.82 µWh cm^−^^2^ and an areal power of 4.53 mW cm^−^^2^. Post‐mortem analysis provided an in‐depth understanding of the stability of the PANI cathode with the PEDOT additive layer, demonstrating the structural and morphological stability of the PANI cathode even after extensive cycling. This study effectively improves the storage capabilities of ZIMBs, steering them toward achieving high‐performance, highly secure planar micro‐batteries.

## 2 Results and Discussion

**Figure 1:**
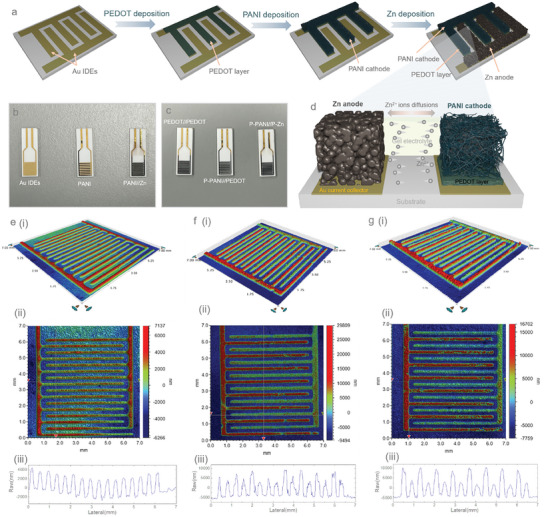
a) Schematic illustration of the processes involved in the fabrication of the P‐PANI//Zn micro‐batteries: The steps include PEDOT coating on one side of Au IDEs, followed by loading PANI onto the same electrode to serve as the cathode, while Zn is loaded onto the opposite IDEs to serve as the anode. b,c) Digital images of the devices at different stages of material loading. d) Schematic demonstrating the Zn^2+^ ions diffusion across micro‐electrodes. During discharge, Zn^2+^ strips from the Zn anode and diffuses into the PANI cathode for the reactions, while the opposite reactions occur during the charging process. 2D and 3D profilometer mappings of the devices including: e) Au IDEs, f) PANI//Zn micro‐battery, and g) P‐PANI//Zn micro‐battery. The graphs below represent the height profiles of the IDEs. The measured thickness of Au IDEs is ≈4 µm. The thicknesses of the PANI cathode and Zn anode in the PANI//Zn micro‐battery are 8.5 and 4 µm, respectively, while the P‐PANI cathode and Zn anode in the P‐PANI//Zn micro‐battery are 11 and 4 µm, respectively.

The preparation process of the P‐PANI//Zn micro‐batteries (PANI deposited on PEDOT‐coated Au cathode and Zn deposited on Au anode) is depicted schematically in **Figure** **1**a. A ceramic chip with a gold integrated electrode (Au IDE) pattern on it was used as the substrate and current collector for the micro‐battery system. PEDOT was first electrodeposited on one side of the pattern, followed by the sequential electrodeposition of PANI on the PEDOT‐coated micro‐electrodes and Zn on the opposite micro‐electrodes (refer to the Experimental Section in the Supporting Information). Zn was chosen as the anode due to its higher theoretical capacities, while PANI served as the cathode, offering a hybrid charge storage mechanism that combines capacitive‐controlled and diffusion‐controlled processes. Figure 1b presents a digital graph of the step‐by‐step process for the PANI//Zn micro‐battery (PANI deposited on Au cathode and Zn deposited on Au anode), and Figure 1c shows the process for the P‐PANI//P‐Zn micro‐battery (PANI deposited on PEDOT‐coated Au cathode and Zn deposited on PEDOT‐coated Au anode). As illustrated in the schematic Figure 1d, in‐plane Zn^2+^ ion diffusion occurs through the gel electrolyte. During discharging, the non‐protonated ─NH─ in PANI is oxidized to ─NH^+^─ and further to ─NH^+^═and ─N═^[^ ^13^, ^14^ ^]^ Zn^2+^ ions strip from the Zn anode during the discharging process, with the reverse reaction occurring during discharging (see further details). Additionally, 2D and 3D profilometer mappings were conducted to explore the surface roughness and estimate the electrode thicknesses of the devices. Figure 1e shows images of the Au IDEs, where the measured thickness of Au IDEs is ≈4 µm. The thickness of the PANI cathode and Zn anode in the PANI//Zn micro‐battery (Figure 1f) is estimated to be 8.5 and 4 µm, respectively (excluding Au IDEs thickness). In the P‐PANI//Zn micro‐battery (Figure 1g), the P‐PANI cathode and Zn anode thicknesses are 11 and 4 µm, respectively (excluding Au IDEs thickness). These images confirm that the materials were successfully loaded onto the Au IDEs uniformly without causing device short circuits (see further investigation of the microelectrodes).

**Figure 2:**
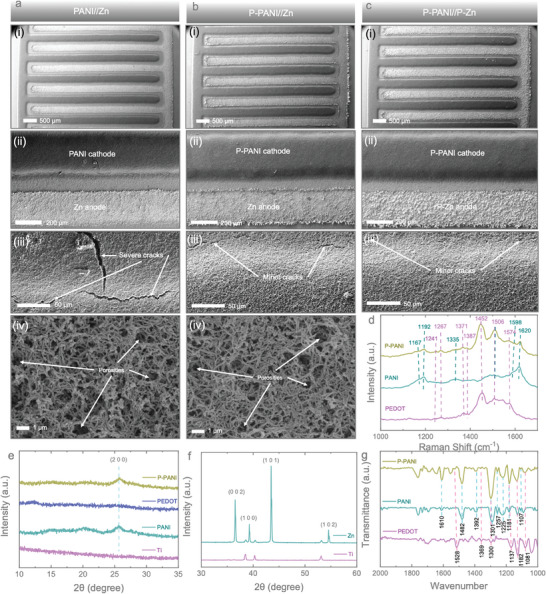
SEM images of a) PANI//Zn micro‐battery, b) P‐PANI//Zn micro‐battery, and c) P‐PANI//P‐Zn micro‐battery from low (i) to high magnification (iv). d) Raman spectra of PEDOT, PANI, and P‐PANI. e)XRD patterns of Ti substrate, PANI, PEDOT, and P‐PANI. f) XRD patterns of Ti substrate and zinc. g) FT‐IR curves of PEDOT, PANI, and P‐PANI.

For further insight into the structural and morphological investigation of the micro‐electrodes, we have extended the study using SEM at different magnifications. **Figure** **2**a shows the SEM images of the PANI//Zn micro‐battery from low (i) to high magnifications (iv). The low magnification SEM images (i and ii) confirm that the loading of the PANI cathode and Zn anode onto Au IDEs is quite uniform without any short circuit issues. However, it is noted that the electrodeposition parameters, including applied voltage and deposition time, have been optimized through a series of experiments, and the optimized conditions are reported in the manuscript (see the experimental section in the Supporting Information). In contrast, severe cracks appear on the PANI loaded onto Au IDEs (see Figure 2a(iii)), which could lead to capacity fading with cycling (discussed in a later section). The deposited PANI maintains nanowire‐like morphologies while preserving porosities, which could be beneficial for the effective diffusion of Zn^2+^ ions, particularly in quasi‐solid‐state gel electrolyte systems. On the other hand, applying a PEDOT layer before loading PANI onto Au IDEs in the P‐PANI//Zn micro‐battery significantly reduces cracking. Only minor cracks appear (Figure 2b), which is beneficial for avoiding cracking and enhancing device stability. No significant change in morphologies and porosities was observed even after loading PANI onto PEDOT‐coated Au IDEs (Figure 2b(iv)). Additionally, the SEM images of the P‐PANI//Zn micro‐battery at different magnifications are shown in Figure 2c. High‐magnification images of Zn deposited on Au IDEs and PEDOT‐coated Au IDEs are provided in the Supporting Information (Figure S1, Supporting Information). Following the application of the PEDOT layer to the Zn anode, the P‐Zn anodes exhibit a closer and more compact arrangement compared to the pristine Zn anode. Moreover, it is noted that while PANI is deposited onto PEDOT‐coated Au IDEs, it generates more current even with the same deposition time as when deposited onto Au IDEs (Figure S2, Supporting Information). This might be due to more PANI loading onto PEDOT‐coated Au IDEs compared to PANI‐loaded Au IDEs, but accurately measuring the active mass loading of PANI onto micro‐electrodes is quite challenging due to the very small device structure, and hence, information related to mass loading is not provided. This increase in surface roughness of the Au IDEs after PEDOT coating likely enhances the loading of PANI.

Figure 2d shows the Raman spectrum of PEDOT, PANI, and P‐PANI deposited on the Au current collector. The purple dotted lines represent the peaks belonging to PANI, while the blue ones correspond to PEDOT. The yellow line indicates a peak position containing different bond vibration signals from both PANI and PEDOT. The characteristic vibrations of both PEDOT and PANI are located between 1000 and 1700 cm^−1^. In the PEDOT spectrum, peaks at 1241 cm^−1^ are produced by C_α_─C_α’_ inter‐ring stretching and C_β_‐H bending, while the C_α_─C_α_ inter‐ring stretching vibration is located at 1267 cm^−1^.^[^ ^15^, ^16^ ^]^ Peaks at 1371 and 1387 cm^−1^ represent C_β_‐C_β_ stretching vibrations.^[^ ^15^, ^17^ ^]^ The C_α_═C_β_ symmetrical bonds are evident at 1452 cm^−1^, and the C_α_═C_β_ asymmetrical bond and in‐plane stretching vibration of carbon atom sp^2^ hybridization are observed at 1506 and 1574 cm^−1^, respectively.^[^ ^16^, ^18^ ^]^ For PANI, C─H bending vibration is denoted by peaks at 1167 cm^−1^. Peaks at 1192, 1506, and 1598 cm^−1^ correspond to C─H vibration in the SQ ring, C═N stretching of quinoid rings, and C─C stretching in the benzene rings, respectively, confirming the existence of the benzene ring.^[^ ^19^, ^20^ ^]^ The C─N^+^ stretching vibration is represented by peaks at 1335 cm^−1^. Peaks at 1620 cm^−1^ signify the stretching vibration of C═C and C─C bonds.^[^ ^19^, ^20^ ^]^ Both PEDOT and PANI peaks can be detected in the P‐PANI coating, confirming the successful preparation and existence of both materials in the layer. For the XRD analysis of the electrodeposited samples, we deposited the samples on Ti foil using the same experimental parameters as those used for loading materials onto Au IDEs. This approach was necessary because capturing XRD data on Au IDEs is not possible due to the small device footprint. Figure 2e shows the XRD pattern of the samples, where the observed peaks for PEDOT and PANI are attributed to diffraction peaks ≈26.0°, corresponding to the (020) plane.^[^ ^21^, ^22^ ^]^ For Zn (Figure 2f), the strong peak ≈26.0° is attributed to the substrate signal, but peaks at 35° and 55° still indicate the presence of zinc. Additional peaks at 36.6°, 39.5°, 43.1°, and 53.4° represent the (002), (100), (101), and (102) crystal planes, respectively.^[^ ^23^ ^]^ In Figure 2g, the FT‐IR spectra of PEDOT, PANI, and P‐PANI are presented within the region of 2000 – 1000 cm^−1^. In the PEDOT spectra, the peak at 1528 cm^−1^ is attributed to the asymmetric stretching mode of the C═C bond, while the peaks at 1369 and 1300 cm^−1^ correspond to the inter‐ring stretching mode of the C─C bonds. The series of peaks at 1182, 1137, and 1081 cm^−1^ display the C─O─C bending vibration in ethylenedioxy.^[^ ^24^, ^25^ ^]^ The presence of PANI is confirmed by another group of peaks. The peaks at 1610 and 1482 cm^−1^ refer to the stretching vibration of the quinoid and the benzene ring, while the peak at 1301 cm^−1^ is due to π‐electron delocalization induced in the polymer through protonation or C─N─C stretching vibration. Two nitrogen‐related peaks appear at 1392 and 1257 cm^−1^, demonstrating the C‐N stretching vibration between benzenoid and quinoid units and the C─N^+^ stretching vibration. The peaks at 1181 and 1107 cm^−1^ represent C─H plane bending vibration and aromatic C─H bending in the plane for the 1,4‐disubstituted aromatic ring, respectively.^[^ ^26^ ^]^

**Figure 3:**
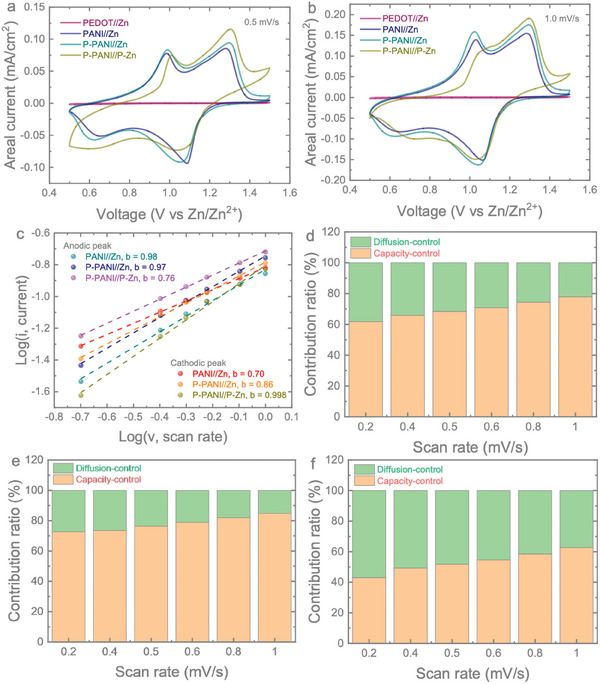
CV curves for PEDOT//Zn micro‐batteries, PANI//Zn micro‐batteries, P‐PANI//Zn micro‐batteries, and P‐PANI//P‐Zn micro‐batteries at scan rates of a) 0.5 mV s^−1^ and b) 1.0 mV s^−1^. c) b‐value fitting curves for the major anodic and cathodic peaks. Diffusion and capacitive contribution bar charts for d) PANI//Zn micro‐batteries, e) P‐PANI//Zn micro‐batteries, and f) P‐PANI//P‐Zn micro‐batteries at scan rates of 0.2, 0.4, 0.5, 0.6, 0.8, and 1.0 mV s^−1^.

The electrochemical performance of the devices was evaluated by soaking them in a 3 M PVA gel electrolyte (Figure S3, Supporting Information, shows the digital picture). **Figures** **3**a,b present the comparative cyclic voltammetry (CV) curves of PEDOT//Zn, PANI//Zn, P‐PANI//Zn, and P‐PANI//P‐Zn micro‐batteries at scan rates of 0.5 and 1.0 mV s^−1^, revealing two pairs of oxidation and reduction peaks corresponding to the multiple redox reactions in PANI.^[^ ^14^ ^]^ The P‐PANI//Zn micro‐batteries exhibit a higher response to areal currents compared to the pristine PANI//Zn micro‐batteries. The P‐PANI//P‐Zn micro‐batteries present higher peak areal currents, but polarisation takes place, and the redox peaks are not as highly reversible as in the other two micro‐batteries (Figures S4a–c, Supporting Information). This could be due to the introduction of PEDOT before coating the Zn anode onto Au IDEs, leading to inefficient reversibility of Zn^2+^ plating/stripping due to reduced conductivity caused by the interlayer between Zn and Au (see further). As a reference, the PEDOT//Zn device shows almost negligible current response, confirming that PEDOT does not influence the electrochemical performance as an electrode material. However, a change in the voltage gap between the cathodic and anodic peaks is observed, which is related to the device's overpotential after the introduction of PEDOT. Specifically, the overpotential refers to the potential difference from the equilibrium potential, caused by charge accumulation at the interface due to the polarization effect. Upon applying the PEDOT layer to the battery system, it becomes evident that polarization is occurring, leading to an increase in overpotential. CV curves of the PEDOT//Zn device at different scan rates (0.2 to 1.0 mV s^−1^) are provided in the Supporting Information (Figure S4d, Supporting Information). The CV curves of PANI//Zn and P‐PANI//Zn micro‐batteries demonstrate similar shapes with minor shifts in redox peaks as the scan rates increase from 0.2 to 1.0 mV s^−1^, while P‐PANI//P‐Zn micro‐batteries suffer from reduced reversibility with increasing scan rates.

For an in‐depth understanding of the charge storage kinetics, we extended the analysis to the charge storage contributions from both diffusion‐controlled and capacitive‐controlled processes. Capacitive‐dominant charge storage offers rapid reversibility and is useful for high‐rate capability batteries. The peak currents (*i_p_*) of CV curves relate to the corresponding scan rates (*v*) as *i_p_* =  *av^b^*, where *a* and *b* are adjustable parameters, and the *b* value indicates the electrochemical charge storage mechanism.^[^ ^27^ ^]^ A value of 0.5 signifies diffusion‐controlled processes, while a value of 1 indicates capacitive‐controlled processes. The calculated *b* values for major peaks of PANI//Zn, P‐PANI//Zn, and P‐PANI//P‐Zn micro‐batteries are shown in Figure 3c. The anodic peaks for the three micro‐batteries are 0.98, 0.97, and 0.76, indicating a predominantly capacitive‐controlled charge contribution. The capacitive‐controlled charge storage process is reduced when PEDOT is added to the Zn anode side in P‐PANI//P‐Zn micro‐batteries. For the reduction peaks, the *b* values are 0.7, 0.86, and 0.99 in the major cathodic peaks. For further quantitative analysis of the charge‐storage contributions, the current at each voltage can be represented as both capacitive‐controlled (*k*_1_ *v*) and diffusion‐controlled (*k*_2_ *v* ^0.5^), thus the current at a specific voltage of CV can be expressed as *i* (*V*) = *i_capacitive_*  +  *i_diffusion_* =  *k*_1_ *v* + *k*_2_ *v* ^0.5^ ⇒ *i*(*V*)/*v* ^0.5^ = *k*_1_ *v* ^0.5^ + *k*_2_^[^ ^26^, ^28^ ^]^ Based on this relationship, we calculated both capacitive‐controlled and diffusion‐controlled contributions to the overall charge storage at different scan rates, as shown in Figures 3d–f. With increasing scan rates, the capacitive contribution increases for all the micro‐batteries. The calculated capacitive contributions at a scan rate of 0.2 mV s^−1^ are 61.70% (for PANI//Zn), 72.62% (P‐PANI//Zn), and 43.09% (P‐PANI//P‐Zn), which increase to 77.91%, 84.80%, and 62.73% at a scan rate of 1 mV s^−1^. P‐PANI//Zn demonstrates a more capacitive‐controlled charge storage contribution compared to the other two, but when both electrodes are coated with PEDOT, the reduced conductivity of the anode might decrease and reduce its participation in the capacitive contribution.

**Figure 4:**
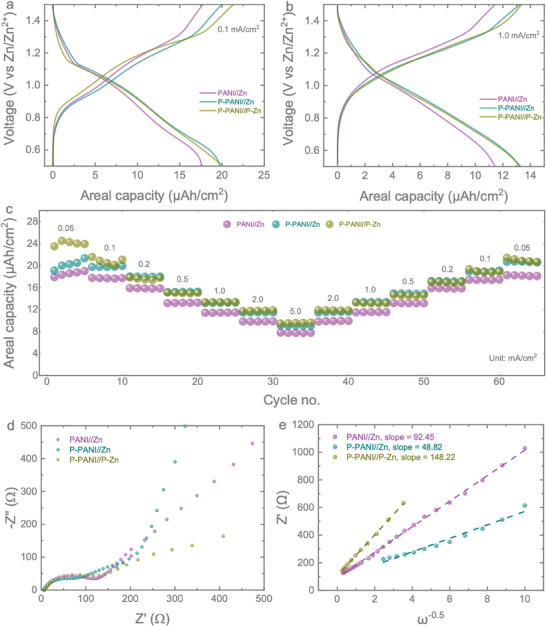
Comparable GCD curves for PANI//Zn micro‐batteries, P‐PANI//Zn micro‐batteries, and P‐PANI//P‐Zn micro‐batteries at a) 0.1 mA cm^−^^2^ and b) 1.0 mA cm^−^^2^. c) Rate tests for PANI//Zn, P‐PANI//Zn, and P‐PANI//P‐Zn micro‐batteries at areal currents of 0.05, 0.1, 0.2, 0.5, 1, 2, and 5 mA cm^−^^2^. d) Nyquist plot of the impedance spectra of PANI//Zn, P‐PANI//Zn, and P‐PANI//P‐Zn micro‐batteries. e) Relative change in Zn^2+^ diffusion coefficients of PANI//Zn, P‐PANI//Zn, and P‐PANI//P‐Zn micro‐batteries.

The next galvanostatic charge‐discharge (GCD) tests of the micro‐batteries were extended at different areal currents ranging from 50 µA cm^−^^2^ to 5 mA cm^−^^2^ over the voltage window of 0.5 to 1.5 V. The GCDs of the micro‐batteries at different currents are provided in the Supporting Information (Figure S5, Supporting Information). Consistent with the CV curves, the P‐PANI//Zn micro‐batteries show a higher areal capacity than the PANI//Zn micro‐batteries. For instance, the areal capacities of PANI//Zn, P‐PANI//Zn, and P‐PANI//P‐Zn at 0.1 mA cm^−^^2^ (**Figure** **4**a) are measured to be 17.64, 19.75, and 20.12 µAh cm^−^^2^, respectively, while the capacities at 1.0 mA cm^−^^2^ (Figure 4b) are 11.43, 13.24, and 13.31 µAh cm^−^^2^. The development in areal capacity confirmed the positive contribution of the PEDOT coating addition. However, it also demonstrates that the double‐sided PEDOT coating in P‐PANI//P‐Zn provides almost the same areal capacity value as the single‐sided coated device (P‐PANI//Zn) but with lower capacity stability over long‐term cycling (see further). Moreover, the P‐PANI//Zn micro‐batteries display excellent rate capability, as depicted in Figure 4c. It is noted that a higher capacitive contribution can lead to improved rate capability, and based on our CV results, both P‐PANI//Zn and PANI//Zn demonstrate a higher capacitive response compared to P‐PANI//P‐Zn batteries. However, the rate test performance shows some variation, with similar areal capacities at high rates when plotted as absolute areal capacity versus cycle number (Figure 4c). Notably, the initial areal capacities of P‐PANI//P‐Zn batteries are higher than those of P‐PANI//Zn and PANI//Zn. The consistency with the CV results becomes clear upon examining the capacity retention plot from the rate test. The P‐PANI//Zn micro‐batteries exhibit areal capacities of 20.50, 19.76, 17.95, 15.20, 13.25, 11.47, and 9.05 µAh cm^−^^2^ at areal currents of 0.05, 0.1, 0.2, 0.5, 1.0, 2.0, and 5.0 mA cm^−^^2^, respectively, while the P‐PANI//P‐Zn samples show capacities of 24.00, 20.12, 17.49, 14.99, 13.33, 11.81, and 9.59 µAh cm^−^^2^. The former maintains a recovered capacity of 20.57 µAh cm^−^^2^, and the latter maintains 20.76 µAh cm^−^^2^, indicating better stability for the P‐PANI//Zn micro‐batteries. This conclusion is further confirmed by the long cycling results (see further). Hence, P‐PANI//Zn micro‐batteries demonstrate higher capacities at each areal current compared to the PANI//Zn micro‐batteries, indicating performance improvements after introducing the PEDOT interfacial layer between PANI and Au IDEs.

To further understand the charge transfer kinetics of the micro‐batteries and the effect of introducing PEDOT as an interfacial layer between the active materials and current collectors, we conducted EIS tests across a frequency range from 10 mHz to 100 kHz with a voltage amplitude of 10 mV. The Nyquist plot, shown in Figure 4d, indicates that the charge transfer resistance of PANI//Zn micro‐batteries is ≈130 Ω. The P‐PANI//Zn micro‐batteries exhibit a lower resistance of ≈100 Ω, while the P‐PANI//P‐Zn samples show a slightly higher resistance of ≈150 Ω. The internal resistance performance further confirms that adding the PEDOT layer on the PANI side can slightly increase conductivity while the layer decreases the conductivity of the Zn coating. Additionally, we extended the relative estimation of relative change in Zn^2^⁺ ion diffusion constants, where the real impedance can be related to the diffusion coefficient as follows: *Z*′ = (*R_L_* + *R_D_*)  + σω^−0.5^ and $D_{zn^{2+}}=\frac{R^{2}T^{2}}{2A^{2}n^{4}F^{4}C^{2}\sigma^{2}}=\frac{K}{\sigma^{2}}$; where (*R_L_* + *R_D_*) is the overall resistance (solution and charge transfer), σ is the Warburg coefficient, ω is the angular frequency, *Z*′ is the real impedance component of the Nyquist plot, $D_{zn^{2+}}$ is the Zn^2^⁺ ion diffusion coefficient (cm^2^ s⁻¹), R is the molar gas constant (8.314 J K⁻¹ mol⁻¹), T is the cell testing temperature in Kelvin (298.13 K), 𝑛 is the number of electrons transferred per electrolyte per monomer unit of PANI, *A* is the active electrode area (cm^2^), 𝐹 is Faraday's constant (96485.3383 C mol⁻¹), and 𝐶 is the Zn^2^⁺ ion molar concentration used in the electrolyte.^[^ ^29^ ^]^ Since the electrode area and electrolyte concentrations during testing are the same, we can consider $k=\frac{R^{2}T^{2}}{2A^{2}n^{4}F^{4}C^{2}}$ the same for all the micro‐batteries. Based on this relationship, we calculated the slopes from the *Z*′ *vs* ω^−0.5^ plot as shown in Figure 4e. The lower slope in P‐PANI//Zn micro‐batteries demonstrates a higher Zn^2^⁺ ion diffusion coefficient compared to the PANI//Zn and P‐PANI//P‐Zn micro‐batteries, where the lowest Zn^2^⁺ ion diffusion coefficient is observed in the P‐PANI//P‐Zn micro‐batteries.

**Figure 5:**
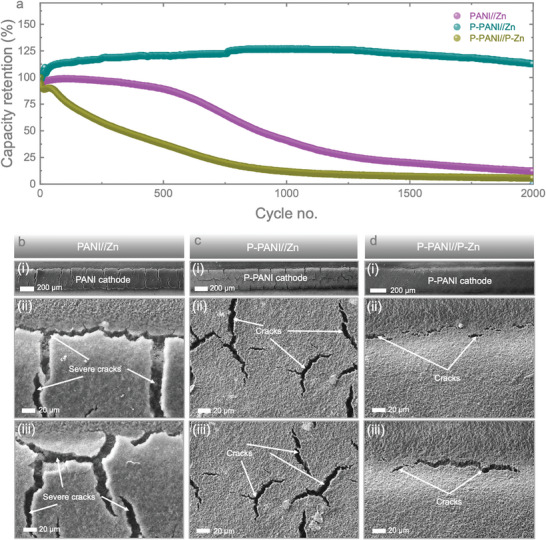
a) Long‐term charge and discharge tests performed at 1 mA cm^−^^2^. SEM images of the cathodes for b) PANI//Zn, c) P‐PANI//Zn, and d) P‐PANI//P‐Zn micro‐batteries at low (i) to high magnification (iii) after 2000 cycles.

To further understand the device stability of the micro‐batteries, we conducted extended cycling tests at 1.0 mA cm^−^^2^ for 2000 cycles, as shown in **Figure** **5**a. The PANI//Zn micro‐battery and the P‐PANI//Zn micro‐battery demonstrate good capacity stability during the first 500 cycles while P‐PANI//P‐Zn started to have a continuous decrease from the beginning, and it remains 5.68% of its capacity at the 2000th cycle. The capacity of PANI//Zn micro‐battery started reducing from the 500th cycle and was retaining only 12.14% by the 2000th cycle. In comparison, the P‐PANI//Zn micro‐battery remains stable, retaining 89.39% of its capacity after 2000 cycles. This confirms that P‐PANI//Zn micro‐batteries demonstrate greater stability compared to the PANI//Zn and P‐PANI//P‐Zn micro‐batteries. This confirms that P‐PANI//Zn micro‐batteries demonstrate greater stability compared to the PANI//Zn and P‐PANI//P‐Zn micro‐batteries. This observation can be attributed to the higher conductivity of the PEDOT layer compared to the PANI electrode. As a result, the PEDOT layer improves reaction kinetics, increasing the electrical conductivity of the cathode material and enhancing efficiency at the cathode side, ultimately boosting the electrochemical performance of P‐PANI//Zn micro‐batteries. However, the PEDOT layer is less conductive than metals, particularly gold and zinc, which results in slower Zn^2^⁺ ion diffusion and increased polarization when the PEDOT interfacial layer is introduced in the P‐Zn anode. This conclusion is further supported by the electrochemical impedance spectroscopy (EIS) test results discussed earlier. To gain further insight into the capacity fading during cycling, we conducted post‐mortem SEM investigations of the micro‐electrodes. Figures 5b–d show the post‐mortem SEM images of the PANI cathodes of PANI//Zn, P‐PANI//Zn, and P‐PANI//P‐Zn micro‐batteries after cycling at different magnifications. It is clear that the PANI cathode in PANI//Zn micro‐batteries undergoes severe cracking, which could lead to capacity fading with cycling. This severe cracking may originate from the initial cracks that appear during PANI deposition onto Au IDEs (discussed earlier, Figure 2a), and crack propagation occurs with cycling. During cracking, some material may detach from the Au IDEs, behaving like dead material and causing capacity fading. In contrast, minor cracks appear in the PEDOT‐coated PANI cathodes in P‐PANI//Zn (Figure 5c) and P‐PANI//P‐Zn (Figure 5d) micro‐batteries. The PEDOT interfacial layer stabilizes the micro‐electrodes, offering better charge storage performance. Therefore, such a strategy is crucial for improving the stability and charge storage performance of the micro‐batteries. Additionally, post‐mortem SEM images of the cycled Zn anode (Figure S6, Supporting Information) show some dendrite formation at the edges of the micro‐electrodes, which is expected as the Zn anode is prone to dendrite growth during cycling. Raman spectroscopy was conducted to examine the composition differences before and after cycling, as shown in Figure S7 (Supporting Information). The peak positions showed minimal shift, but there was a slight change in intensity. This indicates that our material remained quite stable after 2000 charge and discharge cycles, demonstrating the excellent stability of the micro‐batteries.

**Figure 6:**
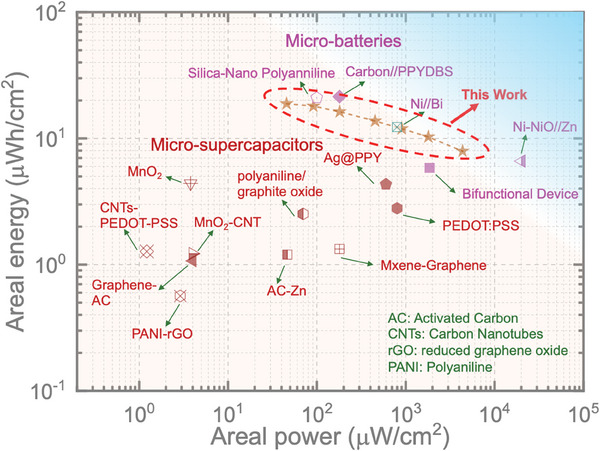
Ragone plot comparing the performance of our micro‐battery with that of reported polymer‐based planar micro‐batteries and high‐performance micro‐supercapacitors.

Moreover, we calculated the areal energies of our micro‐batteries at different areal currents. For instance, the calculated areal energies for PANI//Zn, P‐PANI//Zn, and P‐PANI//P‐Zn micro‐batteries are 17.19, 18.82, and 18.44 µWh cm^−^^2^ respectively at 50 µA cm^−^^2^, and 10.36, 11.93, and 11.83 µWh cm^−^^2^ at 1 mA cm^−^^2^. To further evaluate the performance of our P‐PANI//Zn micro‐battery and compare it with reported micro‐scale devices, we refer to the Ragone plot shown in **Figure** **6**. Our P‐PANI//Zn micro‐battery exhibits impressive charge storage performance, achieving areal powers (at areal energies) of 4.37 mW cm^−^^2^ (at 7.92 µWh cm^−^^2^) and 0.04 mW cm^−^^2^ (at 18.82 µWh cm^−^^2^). These values surpass the performance of most previously reported high‐performance polymer‐based planar micro‐batteries. For instance, a Zn//PANI micro‐battery shows a peak energy density of 5.84 µWh cm^−^^2^ and a peak power density of 1.86 mW cm^−^^2^.^[^ ^30^ ^]^ A Zn//PANI‐GO micro‐battery achieves peak values of 2.52 µWh cm^−^^2^ and 0.07 mW cm^−^^2^.^[^ ^31^ ^]^ The Zn//PANI@Si batteries exhibit a similar energy density of 21.0 µWh cm^−^^2^, but the power density only reaches 0.1 mW cm^−^^2^.^[^ ^32^ ^]^ Additional devices shown in the Ragone plot further illustrate the high efficiency of our P‐PANI//Zn micro‐batteries.^[^ ^31^, ^33^, ^34^, ^35^, ^36^, ^37^, ^38^, ^39^, ^40^, ^41^, ^42^, ^43^ ^]^ Notably, the areal power of our micro‐battery is comparable to or higher than that of planar micro‐supercapacitors, which further supports the overall performance of our micro‐batteries. Moreover, our two‐series integrated micro‐batteries can effectively deliver the required energy to power a moisture sensor, as shown in Figure S8 (Supporting Information).

In summary, we employed a strategy of adding a PEDOT layer to electrodeposit highly stable PANI cathodes, achieving high‐performance planar micro‐batteries. The P‐PANI electrode produced by this method shows remarkable uniformity, capacity, and long‐term stability compared to plain electrodeposited PANI electrodes. The charge storage performance of the P‐PANI as a cathode, paired with Zn as an anode in planar P‐PANI//Zn micro‐batteries, demonstrates an impressive areal capacity of 19.75 µAh cm^−^^2^ at 0.1 mA cm^−^^2^. Additionally, it exhibits an areal energy of 18.82 µWh cm^−^^2^, an areal power of 4.53 mW cm^−^^2^, and stable rateability. The post‐cycling analysis offered a detailed insight into the PANI cathode's stability when combined with the PEDOT additive layer. It emphasized the enduring structural and morphological integrity of the PANI cathode throughout extensive cycling. This research represents a substantial advancement in enhancing the storage capacity of micro‐batteries, aiming for the creation of advanced, secure planar micro‐batteries.

## Acknowledegements

B.D.B. acknowledges support from the EPSRC research grant EP/Y008103/1.

## Conflict of Interest

The authors declare no conflict of interest.

## Supporting information



Supporting Information

## Data Availability

The data that support the findings of this study are available from the corresponding author upon reasonable request.
