# Supplementary material for: Improving Electrochemical Performance in Planar On‐Chip Zn‐ion Micro‐Batteries via Interlayer Strategies
Source: Small. 2024 Oct 14;21(7):2405733. doi: 10.1002/smll.202405733 (PMC11840458; doi:10.1002/smll.202405733)
Supplement: Supplementary file 1 — Supporting Information [file SMLL-21-2405733-s001.pdf]

NANO · MICRO
small

Supporting Information

for *Small*, DOI 10.1002/smll.202405733

Improving Electrochemical Performance in Planar On-Chip Zn-ion Micro-Batteries via Interlayer Strategies

*Yijia Zhu, Nibagani Naresh, Xiaopeng Liu, Jingli Luo, Yujia Fan, Mengjue Cao, Bing Li, Mingqing Wang and Buddha Deka Boruah**

Supporting Information

For

Improving Electrochemical Performance in Planar On-chip Zn-ion Micro-batteries via Interlayer Strategies

Yijia Zhu,[1] Nibagani Naresh,[1] Xiaopeng Liu,[1] Jingli Luo,[1] Yujia Fan,[1] Mengjue Cao,[1] Bing Li,[1] Mingqing Wang,[1] Buddha Deka Boruah[1,*]

[1]Institute for Materials Discovery, University College London, London WC1E 7JE, United Kingdom

Corresponding author: Dr. Buddha Deka Boruah

Email: b.boruah@ucl.ac.uk

Experimental Section

Materials

3,4-ethylenedioxythiophene (EDOT), sodium dodecyl sulfate, H_2SO_4, Na_2SO_4, $ZnSO_4 \cdot 7H_2O$, boric acid, aniline, $Zn(CF_3SO_3)_2$, polyvinyl alcohol (PVA)

Electrodeposition of PEDOT, PANI and Zn: A 0.01 M sodium dodecyl sulfate aqueous solution was prepared, and concentrated H_2SO_4 was added until the H_2SO_4 concentration reached 1 M. EDOT was then introduced into the solution with continuous stirring until the solution turned slightly blue, indicating the formation of the electrolyte. For the preparation of polyaniline (PANI), aniline was added after the H_2SO_4 solution was diluted to 1 M. A mixture of 12.5 g Na_2SO_4, 22.3 g $ZnSO_4 \cdot 7H_2O$, and 2 g boric acid was dissolved in 91 mL deionized water to serve as the electrolyte for Zn deposition.

A commercial gold integrated pattern chip was held with a platinum electrode clip. The electrodeposition processes were conducted using a three-electrode system, with an Ag/AgCl aqueous electrode as the reference electrode and a platinum wire electrode as the counter electrode. The PEDOT layer was first deposited on the pattern by applying 0.9 V for 150 seconds. Subsequently, PANI was deposited using a similar constant voltage process at 0.85 V for 30 seconds. Finally, Zn electrochemical deposition was performed at -40 mA for 8 seconds.

Preparation of PVA gel electrolyte: 1 g of PVA powder was dissolved in 10 mL of deionized water at 85°C. Gradually, 10.9 g of $Zn(CF_3SO_3)_2$ was added to the solution.

Material Characterisation: The morphology and microstructure of the materials prepared in the experiments were characterised using a scanning electron microscope (ZEISS EVO LS15) and profilometer. X-ray diffraction spectroscopy (XRD) detected the samples' structure and composition using Malvern PANalytical Aeris with Cu Kα radiation and Raman spectroscopy (Renishaw inVia, Stockport).

Fabrication of micro-batteries: Two long copper foils, approximately 1.5 mm × 6 cm in size, were attached to the elongated gold electrodes using conductive silver paste. The chip was then positioned in a 1 cm × 1 cm transparent cuvette, and after adding 1 mL of gel electrolyte to

immerse the entire integrated pattern, a piece of parafilm was used to seal the device. To stabilize the battery components, Kapton tape was applied.

Electrochemical tests: A biologic battery tester was used to conduct CV and GCD measurements on PANI-based electrodes. Concurrently, electrodeposited Zn served as the anode, and PVA-$Zn(CF_3SO_3)_2$ gel functioned as the electrolyte. Long cycling tests were performed using NEWARE battery testers, while EIS tests were conducted using the Ivium electrochemical tester.

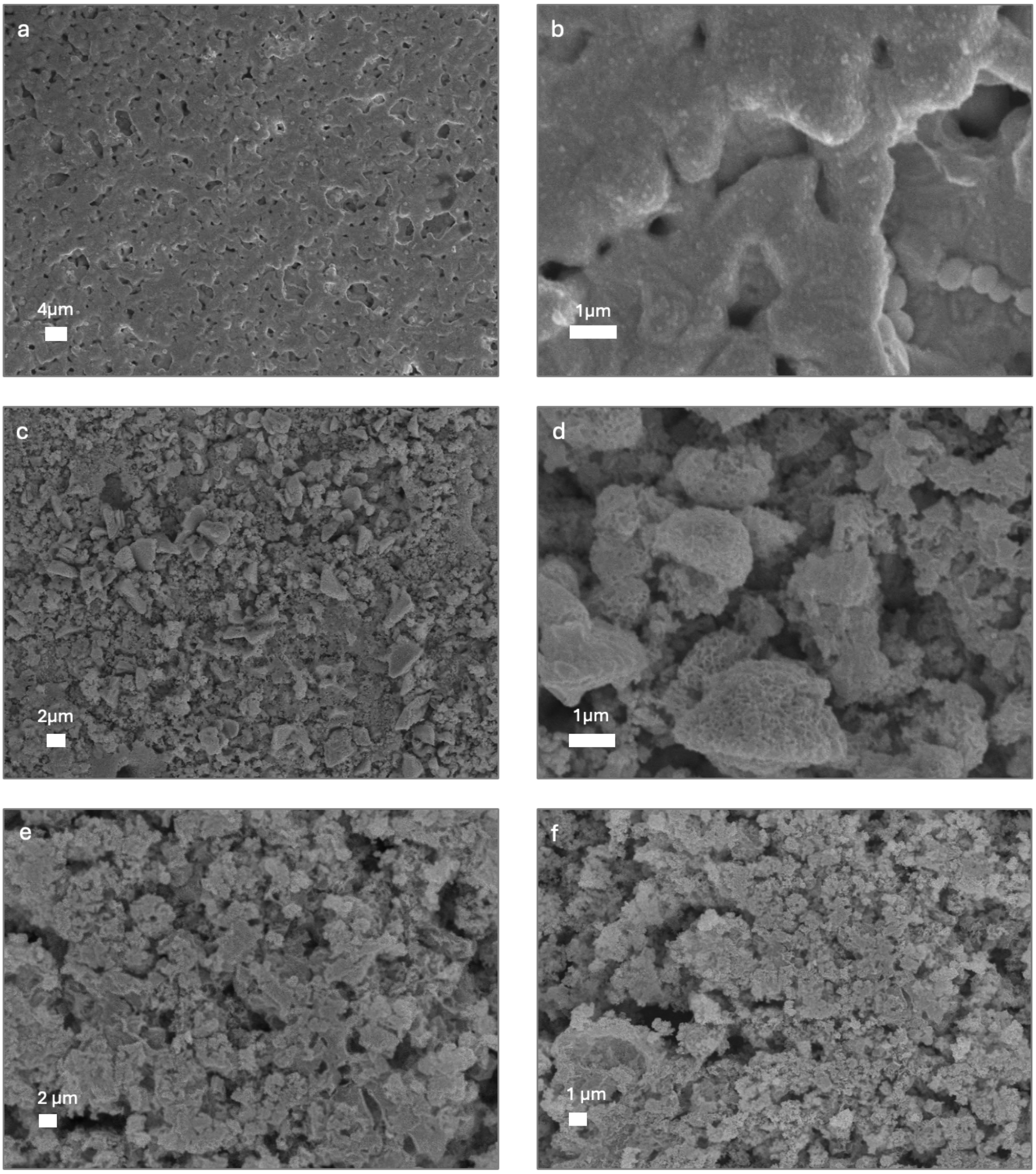

Figure S1. *SEM images of PEDOT on Au IDEs at (a) low and (b) high magnifications. SEM images of Zn on Au IDEs at (c) low and (d) high magnifications. SEM images of Zn on PEDOT at (e) low and (f) high magnifications.*

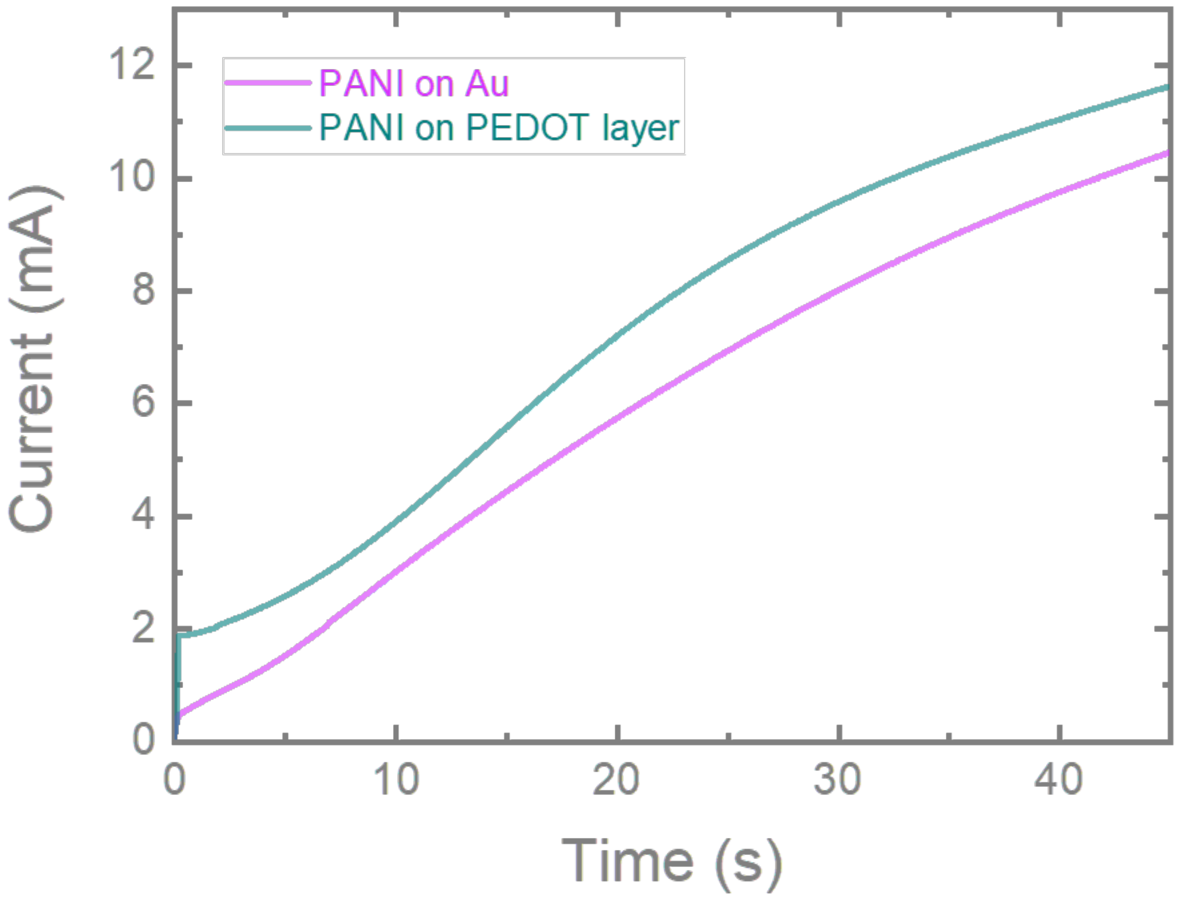

Figure S2. *Current responses of PANI deposition on (a) Au IDEs and (b) PEDOT-coated Au IDEs at a constant voltage of 0.85 V.*

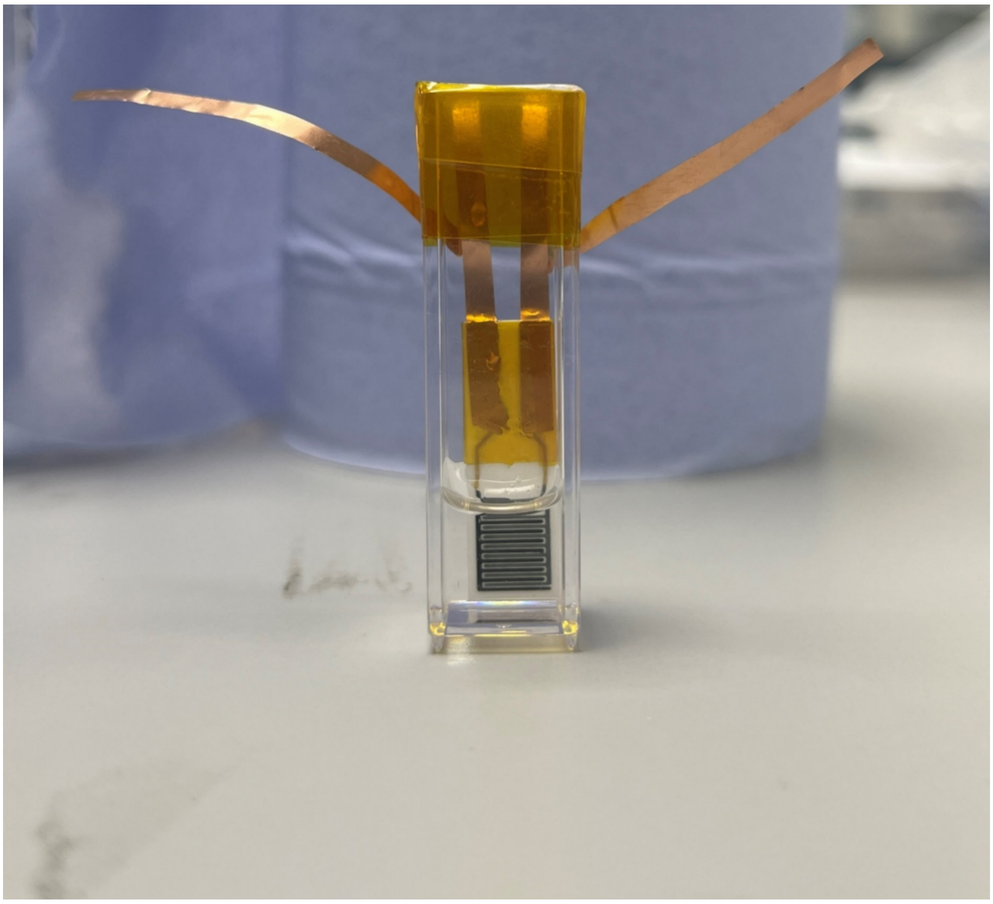

Figure S3. *Digital image of a micro-battery immersed in 3M PVA gel electrolyte for testing.*

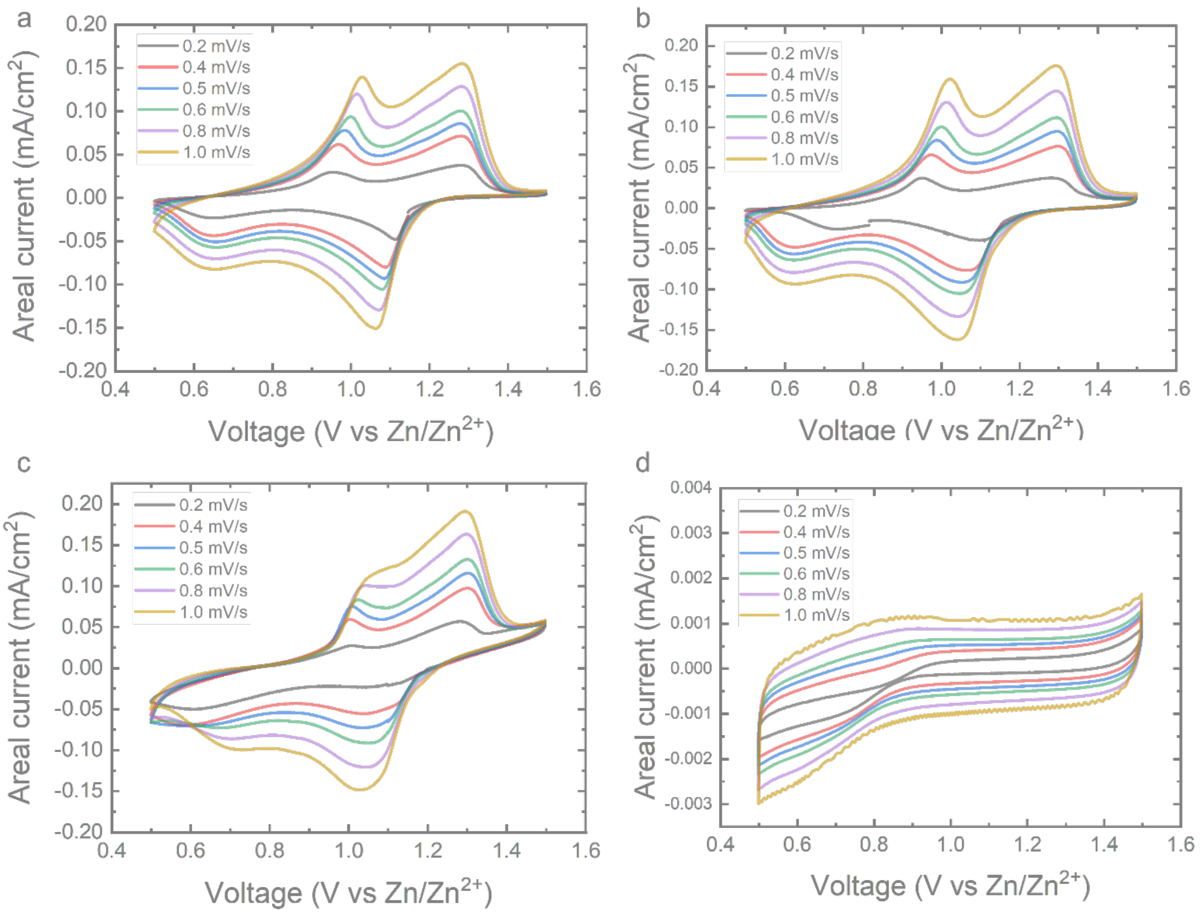

Figure S4. *CV curves for (a) PANI//Zn, (b) P-PANI//Zn, (c) P-PANI//P-Zn, and (d) PEDOT//Zn micro-batteries at 0.2, 0.4, 0.5, 0.6, 0.8, and 1.0 mV/s.*

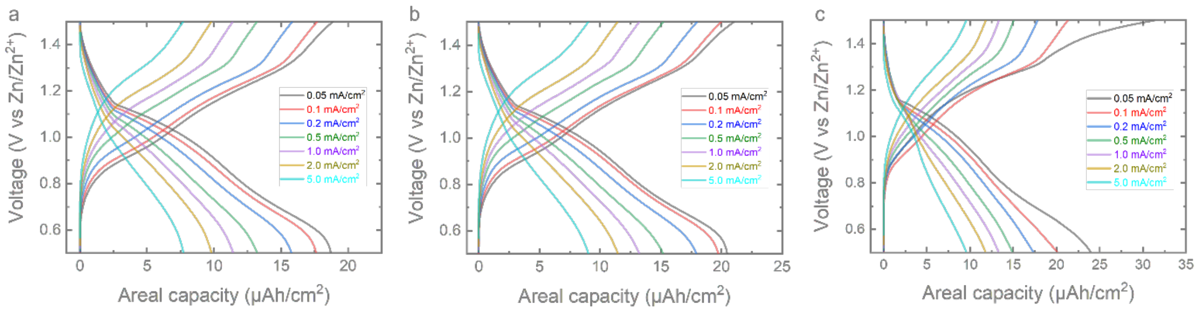

Figure S5. *GCD curves for (a) PANI//Zn, (b) P-PANI//Zn, and (c) P-PANI//P-Zn micro-batteries at 0.05, 0.1, 0.2, 0.5, 1, 2, and 5 mA/cm^2.*

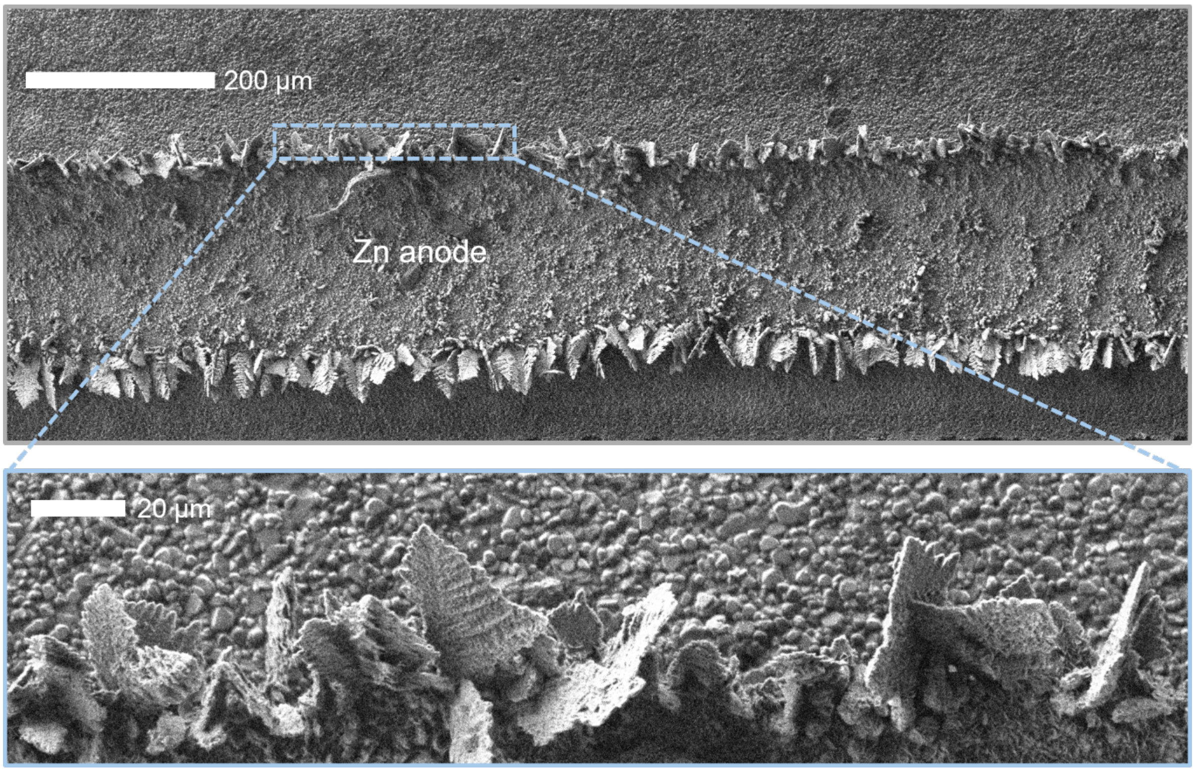

Figure S6. *Post-mortem SEM images of the Zn anode after 2000 cycles.*

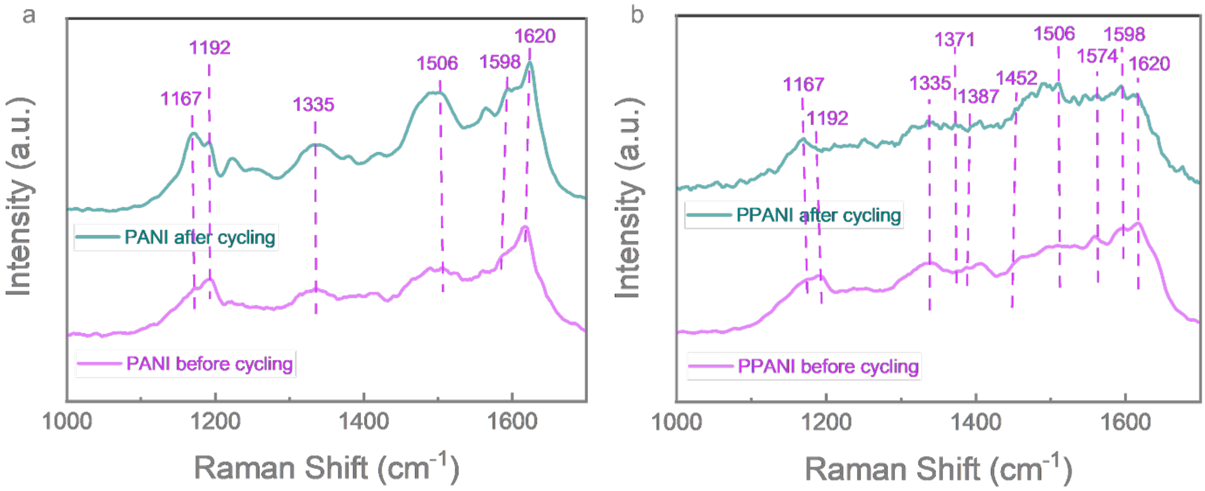

Figure S7. *Raman spectra for (a)PANI and (b)P-PANI before and after cycling.*

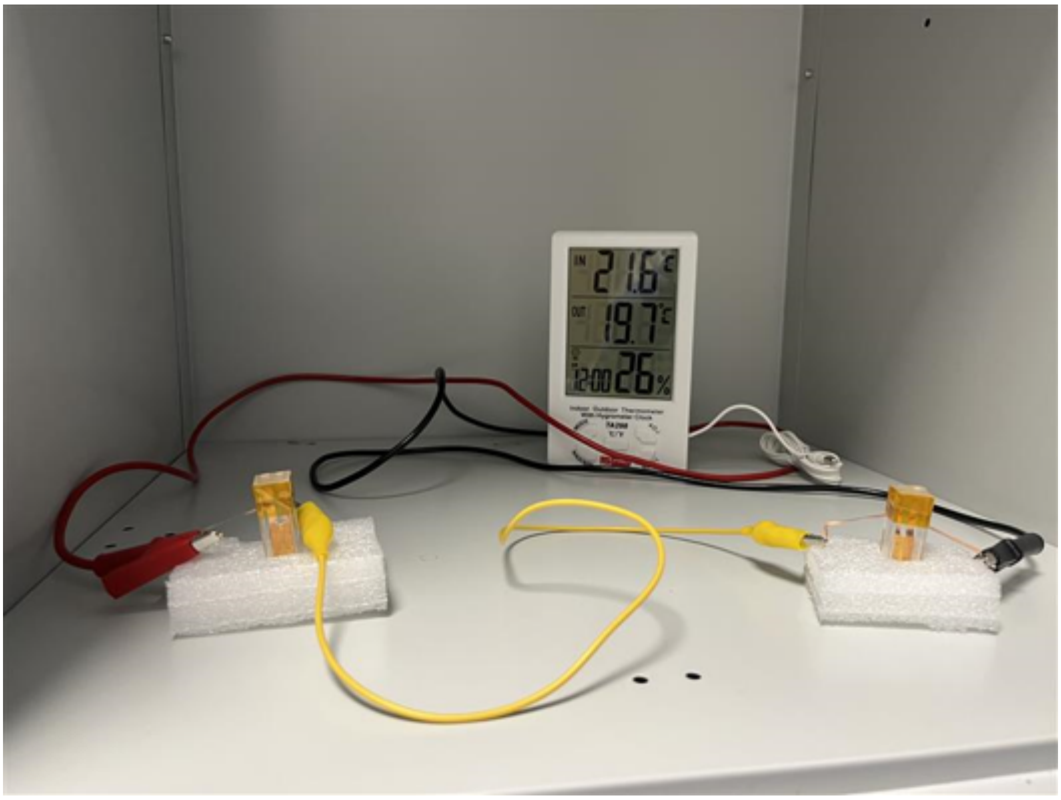

Figure S8. *Digital image of the two-series P-PANI//Zn micro-batteries for powering a moisture sensor.*
